# Medium development beyond production media: Chemically defined media for transfection and single cell cultivation

**DOI:** 10.1186/1753-6561-9-S9-P27

**Published:** 2015-12-14

**Authors:** Tim F Beckmann, Sandra Klausing, Sebastian Püngel, Juliana Coronel, Tim Welsink, Christoph Heinrich

**Affiliations:** 1 Xell AG, 33613 Bielefeld, Germany; 2 InVivo Biotech Services, 16761 Hennigsdorf, Germany; 3 Federal University of Rio de Janeiro, Rio de Janeiro, 21941-596, Brazil

## Background

State-of-the-art medium and feed development has proven its potential for increasing bioprocess efficiency many times. By the application of such advanced chemically defined and animal component-free formulations, in combination with recent cell lines, the yield of mammalian cell cultures was pushed to the binary gram per liter level. In this work, the current progress in designing special application media for transfection and single cell growth is presented. In the context of medium development, these techniques pose specific challenges which differ from production media. However, interest in such specialized products is high. On one side, clone selection is a key factor for robust and effective processes. On the other side, applications for transient transfection range from purposes in R&D, over the production of pre-clinical material to personalized medicine or vaccine production. In terms of upscaling, efficient transient gene expression (TGE) processes require a bifunctional medium supporting both cell growth and transfection.

## Material and methods

The work on a chemically defined and animal component-free (ACF) medium for single cell growth was performed in 96 well plates in comparison to serum containing medium. Single cell growth was analyzed using a Cellavista. A proof of concept for clone selection was performed with different CHO cells including upscaling from 96-well plates to shake flasks. Labile recombinant protein expressed by isolated subclones was quantified by ELISA and activity assay. For transient transfection, the potential of the developed formulation was demonstrated using various mammalian cell lines (human and CHO). The transfection efficiency was evaluated using a polycationic transfection reagent and a GFP expression plasmid. Additionally, the expression of an IgG1 antibody was investigated for a HEK cell line by batch, fed-batch and pseudo-perfusion performing shaking flask cultivations after transient transfection in fresh medium. Quantification of monoclonal antibody titers was realized by protein A HPLC. The setup for transfection varied in detail, but in general cells were transfected at densities of 3·106 cells/mL using a polycationic transfection reagent. 2 pg DNA/cell were used in transfections with plasmids harboring genes for the expression of GFP or an IgG1. Culture volumes during transfection ranged from 4 mL to 1000 mL.

## Results

The performed proof of concept already illustrates the capability of the current medium stage of the single cell medium. Different single cells were successfully expanded from 96 well plates, to 24 well plates, T-flasks up to shaking flasks. The differences in final growth performance and productivity of the selected clones exhibited the heterogeneity within the original population which was used for limited dilution. Regarding growth performance in batch cultivation, the peak cell densities of eight exemplary subclones ranged from 1.9·106 cells/mL to 8.5·106 cells/mL. Product yield and activity of the expressed labile protein varied by more than factor 3. For the used development stage of the animal component-free cloning medium, cloning efficiency was still higher with serum addition. Nevertheless, the use of an animal component-free cloning medium is advantageous since the transfer to final ACF production medium is significantly eased.

**Figure 1:**
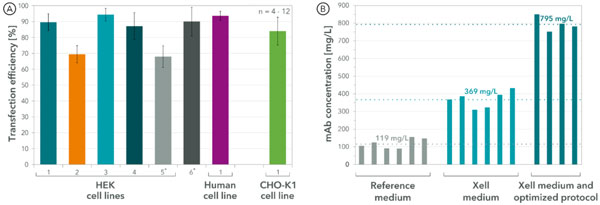
**Overview of transfection efficiency (A) achieved using the developed medium**. Although designed for HEK cell lines, the medium also supports high transfection rates for another human cell line as well as for CHO-K1. Furthermore, transient gene expression was evaluated by monoclonal antibody yields (B). Results obtained using a well know commercially available medium recommended for TGE were compared to titers achieved with the developed Xell medium.

Referring to transient transfection, the developed HEK transfection medium supports stable cell growth in seed train as well as high cell densities (up to 16·106 cells/mL) and viability in batch cultivation. Using fresh medium at the time of gene delivery, efficiencies in the range of68% -94% for various HEK cell lines and 75% - 92% for one evaluated CHO cell line were achieved (see Figure 1A). A successful small scale pseudo-perfusion cultivation shows the feasibility of this process strategy for transient gene expression and is simply scaled-up to bioreactors. Such perfusion processes are highly interesting for labile products, e.g. specific enzymes. Furthermore, monoclonal antibody titers of 90 -150 mg/L were measured with a commercially available reference medium that is recommended for transient gene expression in such cell lines. The application of our developed Xell medium resulted in elevated titers of 310 - 430 mg/L, which is equivalent with an increase by factor 3.1 on average. Design of experiment (DOE) based optimization of the protocol with addition of a specially tailored feed after transfection further increased the yield to 750 - 850 mg/L. Compared with the commercially available reference this is an improvement by factor 6.7. An overview of these results is illustrated in Figure 1B.

## Acknowledgements

Parts of this work were financially supported by the German Federal Ministry of Education and Research - BMBF. Responsibility for the content lies with the author.

